# Ultra-sensitive hybrid diamond nanothermometer

**DOI:** 10.1093/nsr/nwaa194

**Published:** 2020-08-28

**Authors:** Chu-Feng Liu, Weng-Hang Leong, Kangwei Xia, Xi Feng, Amit Finkler, Andrej Denisenko, Jörg Wrachtrup, Quan Li, Ren-Bao Liu

**Affiliations:** Department of Physics, The Chinese University of Hong Kong, Hong Kong, China; Department of Physics, The Chinese University of Hong Kong, Hong Kong, China; Department of Physics, The Chinese University of Hong Kong, Hong Kong, China; Department of Physics, The Chinese University of Hong Kong, Hong Kong, China; 3rd Institute of Physics and Center for Applied Quantum Technologies, University of Stuttgart, 70569 Stuttgart, Germany; 3rd Institute of Physics and Center for Applied Quantum Technologies, University of Stuttgart, 70569 Stuttgart, Germany; 3rd Institute of Physics and Center for Applied Quantum Technologies, University of Stuttgart, 70569 Stuttgart, Germany; Max Planck Institute for Solid State Research, 70569 Stuttgart, Germany; Department of Physics, The Chinese University of Hong Kong, Hong Kong, China; The Hong Kong Institute of Quantum Information Science and Technology, The Chinese University of Hong Kong, Hong Kong, China; Department of Physics, The Chinese University of Hong Kong, Hong Kong, China; The Hong Kong Institute of Quantum Information Science and Technology, The Chinese University of Hong Kong, Hong Kong, China

**Keywords:** nano-thermometry, diamond, nitrogen-vacancy center, quantum sensing, magnetic nanoparticle

## Abstract

Nitrogen-vacancy (NV) centers in diamond are promising quantum sensors because of their long spin coherence time under ambient conditions. However, their spin resonances are relatively insensitive to non-magnetic parameters such as temperature. A magnetic-nanoparticle-nanodiamond hybrid thermometer, where the temperature change is converted to the magnetic field variation near the Curie temperature, were demonstrated to have enhanced temperature sensitivity ($11{\rm{\,\,mK\,\,H}}{{\rm{z}}^{ - 1/2}}$) (Wang N, Liu G-Q and Leong W-H *et al*. *Phys Rev X* 2018; 8: 011042), but the sensitivity was limited by the large spectral broadening of ensemble spins in nanodiamonds. To overcome this limitation, here we show an improved design of a hybrid nanothermometer using a single NV center in a diamond nanopillar coupled with a single magnetic nanoparticle of copper-nickel alloy, and demonstrate a temperature sensitivity of $76{\rm{\,\,\mu K\,\,H}}{{\rm{z}}^{ - 1/2}}$. This hybrid design enables detection of 2 mK temperature changes with temporal resolution of 5 ms. The ultra-sensitive nanothermometer offers a new tool to investigate thermal processes in nanoscale systems.

## Teaser

A combination of a magnetic nanoparticle and a single nitrogen-vacancy center in a diamond nanopillar makes a nano-thermometer with a record sensitivity 76 ÂμK Hz^-1/2^ under ambient condition, adding a tool to many studies of nanoscale thermal processes.

## INTRODUCTION

Nanoscale temperature measurement with high sensitivity is important in investigation of many phenomena such as thermal mapping of nano-/micro-electronics [1], thermoplasmonics of nanoparticles [2], chemical reactions in nanoliter volume [3] and thermal processes in live systems [4–6]. Various measurement protocols have been developed to probe the thermal dynamics on the nanoscale. Optical thermometers convert local temperature variation to changes of optical lifetimes [7], fluorescence intensities [8], Raman shifts [9] or emission spectra [10]. Being a non-contact and convenient method, optical-based nanothermometers, like fluorescence proteins [11], dyes [7] and rare-earth nanoparticles [12], have been proposed and demonstrated for temperature detection under various conditions. However, this method has a relatively low sensitivity (typically $1\,\,{\rm{K\,\,H}}{{\rm{z}}^{ - 1/2}}$) [13–15], and some optical sensors are subject to artifacts induced by the local environments of the sensors, such as refractive indices and pH values [16]. Electronic temperature measurements, such as scanning thermal microscopy and superconducting quantum interference devices (SQUID), have high spatial resolution and high sensitivity ($\sim 1{\rm{\,\,\mu K\,\,H}}{{\rm{z}}^{ - 1/2}}$) [17,18], but they require extreme operating conditions and are subject to contact-related artifacts.

The recent development of diamond-based thermometers provides a promising alternative [19–21]. Nitrogen-vacancy (NV) centers in diamond have long spin coherence time under ambient conditions [22]. Their spin resonance frequencies shift with the environmental temperature [23], which is robust against artifacts from local environments. With photo-stability of NV centers [24], high thermal conductivity [25] and bio-compatibility of the diamond material [26,27], diamond-based thermometers are a potential candidate for temperature sensing in complex systems without requirement for extreme operating conditions. However, the temperature dependence of NV center spin transition frequencies ($dD/dT\,\, \approx - 74\,\,{\rm{kHz\,\,}}{{\rm{K}}^{ - 1}})$ is relatively small. Thus, there arises the idea of hybrid diamond thermometers [28,29], in which the temperature change is transduced to a magnetic signal to be detected by the NV center spins. A hybrid nanothermometer composed of a single copper-nickel alloy magnetic nanoparticle (MNP) and a diamond nanocrystal with ensemble NV centers [28] were demonstrated to have a sensitivity as high as $11\,\,{\rm{mK\,\,H}}{{\rm{z}}^{ - 1/2}}$, near the Curie temperature of the magnetic nanoparticle, where a small temperature change leads to a large magnetic field change as a result of the critical magnetization. However, the sensitivity of this hybrid nanothermometer was limited by the short coherence time of ensemble NV centers in nanodiamonds as well as the optically detected magnetic resonance (ODMR) linewidth broadening from the large gradient of the magnetic field from the magnetic nanoparticle. To overcome this limitation, here we constructed a hybrid nanothermometer employing a single NV center in a diamond nanopillar and a single copper-nickel alloy nanoparticle. This design has the following advantages: the spin coherence time of the single NV center is much longer than those in nanodiamonds and the field gradient induced broadening of ensemble NV centers in nanodiamond is eliminated [28]. Although the photon count rate of a single NV center is lower than those of ensemble NV centers in nanodiamonds, the pillar waveguide configuration largely enhances the photon collection efficiency [30]. We constructed the hybrid nanothermometer by placing the magnetic nanoparticle close to the diamond nanopillar via nano-manipulation based on atomic force microscopy (AFM). Such a hybrid nanothermometer has a temperature sensitivity of 76 ${\rm{\mu K\,\,H}}{{\rm{z}}^{ - 1/2}}$, enabling detection of 2 mK temperature changes with temporal resolution of 5 ms. To the best of our knowledge, this is the most sensitive nanothermometer working under ambient conditions. Employing this hybrid sensor, we monitored the temperature changes of a laser heating process and environmental temperature fluctuations, as well as thermal dissipation near the sensor when additional heating to the system was induced by controlling the current passing through the microwave antenna. This ultra-sensitive hybrid nanothermometer offers an opportunity to study fast thermal processes in nanostructures and/or in living systems.

## RESULTS

**Figure 1.**
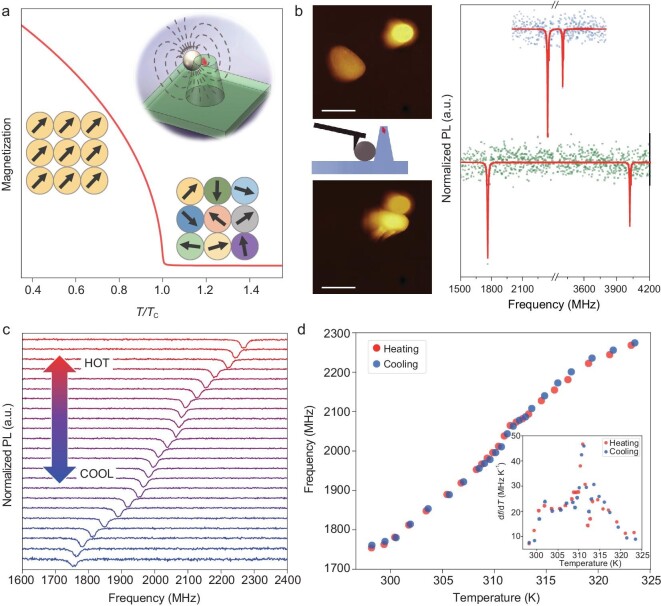
Design of a hybrid nanothermometer composed of a single magnetic copper-nickel alloy nanoparticle and a single nitrogen-vacancy (NV) center in a diamond nanopillar. (a) Simulation of the magnetization M of a copper-nickel alloy nanoparticle as a function of temperature under a magnetic field of 100 Gauss. The inset illustrates the configuration of the hybrid nanothermometer. (b) Atomic force microscopy (AFM) image of the copper-nickel alloy magnetic nanoparticle (MNP) and the diamond nanopillar before the nanomanipulation (upper graph) and after the nanomanipulation (lower graph), and the corresponding optically detected magnetic resonance (ODMR) spectra of the single NV center before and after nanomanipulation (dots being measurement data and lines the double Lorentzian peak fitting). Scale bar is 1 ${\rm{\mu m}}$. (c) ODMR spectra of the hybrid nanothermometer at different environmental temperatures (from 298 K to 324 K from bottom to top). (d) ODMR frequency shifts in the heating (red) and cooling (blue) processes. The inset shows the temperature susceptibility of the hybrid nanothermometer, which has the maximum $\textit{df}/\textit{dT}{\sim}47\,\,{\rm{MHz\,\,}}{{\rm{K}}^{{\rm{ - 1}}}}$ (at 311 K).

The hybrid diamond nanothermometer is composed of a single NV center in a diamond pillar and a copper-nickel alloy MNP, as illustrated in the inset of Fig. 1a. The ground state of an NV center is a spin triplet. The simplified spin Hamiltonian can be written as

(1) \begin{equation*}H = D{{\bf{S}}^2} + {\gamma _{\rm{e}}}{\bf{B}} \cdot {\bf{S}}, \end{equation*}

where ${\mathbf{S}}$ is the spin operator, ${\mathbf{B}}$ is the external magnetic field, $D \approx 2.87\,\,{\rm{GHz}}$ is the zero-field splitting between the ${m_s} = \,\,0$ and the ${m_s} = \,\, \pm 1$ states, and the electron gyromagnetic ratio ${\gamma _{\rm{e}}} = \,\,2.8\,\,{\rm{MHz\,\,Gaus}}{{\rm{s}}^{ - 1}}$. The transition frequencies between different spin states can be measured by ODMR spectroscopy using the spin-dependent fluorescence and resonant microwave manipulation of the spin. Unlike conventional diamond thermometry based on the temperature dependence of *D*, which has a susceptibility of $dD/dT \approx - 74\,{\rm{kHz}}\,{{\rm{K}}^{ - 1}}$, the hybrid nanothermometer measures the magnetization change of the MNP induced by temperature variation [28]. Near the critical point of the MNP, the temperature susceptibility is large and hence a high temperature sensitivity can be achieved. Figure 1a shows a simulated demagnetization curve when an MNP undergoes ferromagnetic-paramagnetic transition under a small external magnetic field (100 Gauss). The magnetization of the MNP changes drastically when the temperature approaches the Curie point (${T_{\rm{C}}}$). The magnetic field from the MNP induces the Zeeman splitting between the ${m_s} = \,\, - 1$ and ${m_s} = \,\, + 1$ states of the NV center, which can be measured via ODMR spectroscopy. Using a single NV center in a diamond nanopillar has several advantages over the previous hybrid configuration [28]. First, single NV centers in diamond have longer coherence times than ensemble NV centers in nanodiamonds. In our experiments, the selected NV center has ${\rm{a\,\,}}$dephasing time $T_2^*\sim1.5{\rm{\,\,\mu s}}$ (see Supplementary Fig. 2 for details of optical and spin properties of the NV center). As a comparison, the typical dephasing time of NV centers in nanodiamonds is $\sim100{\rm{\,\,ns}}$ [31]. Second, although the fluorescence intensity of a single NV center is lower than that of ensemble NV centers in nanodiamonds, the waveguide effect of the nanopillar structure makes the emission more directional and therefore enhances the fluorescence collection efficiency [30]. Third, the large inhomogeneous broadening of the ODMR of ensemble NV centers in nanodiamonds from the gradient of the magnetic field from MNPs is absent in the case of single NV centers.

The hybrid nanothermometer was constructed by nanomanipulation in an AFM setup. A key factor of the hybrid nanothermometer is effective coupling between the MNP and the NV center, which strongly depends on their distance and relative orientation. Figure 1b shows an example of nanomanipulation. A much larger splitting of the ${m_s} = \,\, \pm 1$ states appeared in the ODMR spectra when the MNP was pushed closer to the diamond nanopillar. Apart from the coupling strength between the MNP and the NV center, the working range of the hybrid sensor is tens of Kelvin below the Curie temperature. The Curie temperature can be designed by tuning the chemical composition of the copper-nickel alloy nanoparticle. Thus, the hybrid sensor can have a broad working range from cryogenic temperatures to about 600 K [28].

To characterize the temperature response of the magnetization of the copper-nickel alloy MNP, we measured the magnetic field at the NV center using continuous-wave ODMR spectroscopy. The environmental temperature was controlled by a ceramic heater and calibrated by monitoring the *D* shift of a reference NV center that is far from any MNP (therefore under zero magnetic field) (see Supplementary Fig. 3). After the temperature calibration, a magnetic field of $192{\rm{\,\,Gauss}}$ was applied to enhance the local magnetic field generated by the MNP. Figure 1c plots a series of ODMR spectra of the hybrid nanothermometer at different temperatures. The resonance dips indicate the transition between the ${m_s} = {\rm{\,\,}}0$ and ${m_s} = \,\, - 1$ states of the NV center spin. The spin resonance frequencies at different temperatures are plotted in Fig. 1d. With increasing environmental temperature, the resonance frequency splitting was reduced via thermal demagnetization of the MNP. The magnetization of the MNP presented a sensitive response. The inset in Fig. 1d summarizes the temperature susceptibility $\textit{df}/\textit{dT}$ of the NV center spin resonance frequency (${m_s} = \,\, - 1$ state). At $38^\circ {\rm{C}}$, the susceptibility reached its maximum of $47{\rm{\,\,MHz\,\,}}{{\rm{K}}^{ - 1}}$. Compared to the temperature-dependent *D* shift of an NV center spin, $dD/dT \approx - 74\,\,{\rm{kHz}}\,{{\rm{K}}^{ - 1}}$, the temperature susceptibility of the hybrid nanothermometer is enhanced approximately 600-fold. Furthermore, the magnetization and demagnetization of this MNP are reversible under the external magnetic field ($192{\rm{\,\,Gauss}}$) alignment, as evidenced by the overlap between the temperature responses during the heating and cooling processes (Fig. 1d). The reversibility and chemical stability of the hybrid nanothermometer were verified by repeating heating/cooling measurements on the same hybrid sensor at different times (see Supplementary Fig. 4).

**Figure 2.**
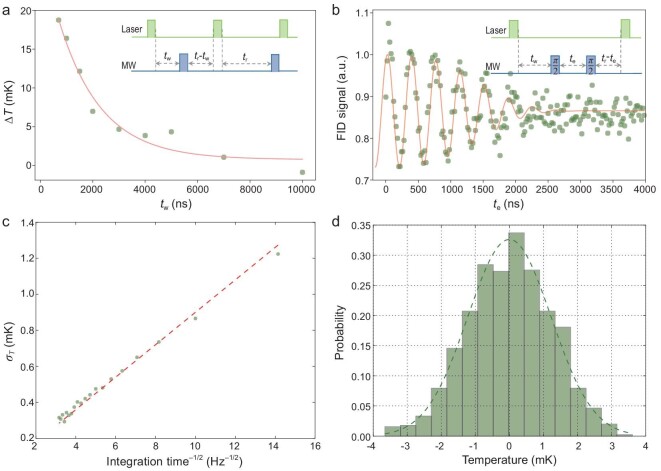
Sensitivity of the hybrid nanothermometer. (a) Cooling curve of the copper-nickel alloy MNP after the laser was turned off. Inset: pulse sequence for measuring the cooling dynamics of the MNP in the hybrid nanothermometer. (b) Free induction decay (FID) of the NV center spin in the hybrid nanothermometer. The inset shows the pulse sequence. The delay time ${t_w}$ between the initialization laser and the microwave pulse sequence was chosen to be the cooling time of the MNP (${t_w}$ = 1500 ns). (c) Dependence of the temperature standard deviation on data integration time using FID real-time measurement. The shot-noise limited sensitivity is derived from the slope of the fitting curve (red dashed line). (d) A typical histogram of temperature measured in a period of 30 seconds (with sampling time 5 ms).

For high-precision temperature measurement, it is important to exclude the laser heating effect. In conventional optical-based nanothermometers, laser heating on the thermometers induces a local temperature increase [32], which then complicates measurement of the environmental temperature. Laser heating also occurs with our hybrid nanothermometer; however, the pulsed ODMR protocol allows a large reduction of this effect as the laser can be turned off during the spin evolution period in pulsed measurement. The protocol of the pulsed measurement is as follows (see inset of Fig. 2a): First a laser pulse was applied to initialize the spin; then after a waiting time ${t_{\rm{w}}}$ a microwave was applied; and finally a laser pulse was applied after time ${t_{\rm{r}}} - {t_{\rm{w}}}$ to read out the spin state (which also serves to initialize the spin for the next shot of measurement). The interval (${t_{\rm{r}}}$) between laser pulses was kept constant to maintain the heating effect for various waiting times (${t_{\rm{w}}}$) taken between the laser and microwave pulses. To understand the cooling dynamics in the hybrid nanothermometer, we carried out pulsed ODMR measurements with different waiting times ${t_{\rm{w}}}$. The environmental temperature was set at $38^\circ {\rm{C}}$, where the temperature susceptibility attains the maximum $df/dT\,\, = \,\,47\,\,{\rm{MHz\,\,}}{{\rm{K}}^{ - 1}}$. Considering that the environmental temperature would change with millikelvin scale during long-term measurement, a reference ODMR measurement with waiting time ${t_{\rm{w}}} = {\rm{\,\,}}10{\rm{\,\,\mu s\,\,}}$between the laser and microwave pulses was performed simultaneously to calibrate the spin resonance frequency drift resulting from long-term temperature fluctuation. Figure 2a shows the temperature dynamics of the hybrid sensor as a function of the waiting time ${t_{\rm{w}}}$. The pulsed laser excitation (300 ns) of the hybrid nanothermometer induced a local temperature increase of about 20 mK. Such an increase of temperature is several times larger than temperature fluctuations of interest in, for example, nanoelectronics and biological systems [4,17]. After the laser was turned off, the local temperature decayed exponentially and recovered to the environmental temperature within a timescale of $\sim1.5{\rm{\,\,\mu s}}$. Thus, the laser heating effect can be largely reduced by choosing a waiting time ${t_{\rm{w}}}{\rm{\,\,}} \ge 1.5{\rm{\,\,\mu s}}$. In the following experiments, we chose ${t_{\rm{w}}} = {\rm{\,\,}}1.5{\rm{\,\,\mu s}}$ to reduce the laser heating effect while still having a reasonable measurement duty ratio.

**Figure 3.**
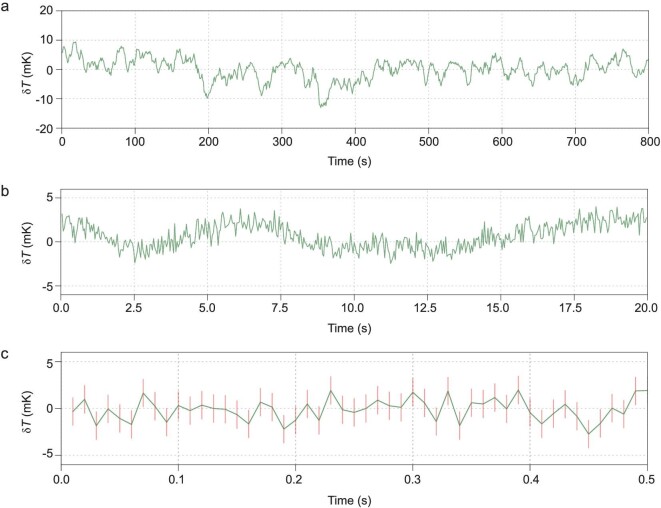
Real-time monitoring of local thermal dynamics. (a–c) Environmental temperature fluctuation measured by the hybrid nanothermometer with various data integration times (0.5 s, 40 ms and 10 ms, in a, b, and c, respectively).

To determine the temperature sensitivity of the hybrid nanothermometer, free-induction decay (FID) of the NV electron spin was measured (Fig. 2b). The pulse sequence of the FID measurement (inset of Fig. 2b) was modified to reduce the laser heating effect ( ${t_{\rm{w}}} = {\rm{\,\,}}1.5{\rm{\,\,\mu s}}$), while the interval between the laser pulses was kept constant so that the total laser power applied to the sample was the same for different FID times (see Supplementary Fig. 5 for comparison of the FID signal with and without the pulse modification). At the maximum temperature susceptibility point ($38{\rm{^\circ C}}$), the sensitivity of the hybrid nanothermometer is estimated to be 76 ${\rm{\mu }}$K Hz^−1/2^ (see Methods for details of the sensitivity estimation). Consistent results were obtained from two other hybrid sensors, revealing the robustness and reproducibility of our hybrid quantum thermometer design (see Supplementary Fig. 6). To further verify that the sensitivity was shot-noise limited, we carried out real-time FID measurement with an optimized waiting time of $983{\rm{\,\,ns}}$ (where we had the maximum resonance frequency susceptibility of the FID signal, see Methods for details). The linear dependence of the temperature accuracy (defined as the standard deviation of the temperature measurements ${\sigma _T}$) on the inverse square root of integration time (see Fig. 2c) indicates that the sensitivity is shot-noise limited, with a shot-noise limited sensitivity in the real-time measurement of about 87 ${\rm{\mu }}$K Hz^−1/2^. With such high sensitivity, our hybrid nanothermometer provides the capability to measure millikelvin temperature dynamics with a temporal resolution of a millisecond. For example, Fig. 2d is a histogram of the uncertainty of the measured temperatures with a sampling time of 5 ms. The distribution presents Gaussian statistics with a standard deviation of 1.5 mK. As a comparison, the previous version of the nanodiamond-based hybrid sensor (with sensitivity of $11\,\,{\rm{mK\,\,H}}{{\rm{z}}^{ - 1/2}}$) [28] would need 50 seconds of measurement time to achieve the same precision. The almost-two-orders-of-magnitude enhanced sensitivity would be beneficial for a wide range of applications, especially in measuring millikelvin temperature change (induced by environmental fluctuation, laser heating, or dissipation from micro/nanostructures) with high temporal resolution. To demonstrate the hybrid nanothermometer as a powerful temperature monitor, we performed environmental temperature tracking by measuring environmental temperature dynamics at various timescales (Fig. 3a–c). The temperature fluctuation had maximum amplitudes of $ \pm 10\,\,{\rm{mK}}$, $ \pm 5\,\,{\rm{mK}}$ and $ \pm 2\,\,{\rm{mK}}$, at timescales of 100, 1 and 0.1 seconds, respectively.

**Figure 4.**
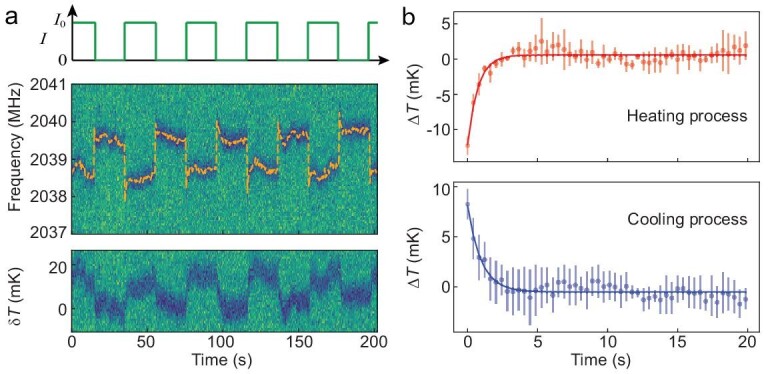
Heat dissipation dynamics in the hybrid nanothermometer under pulsed heating. (a) Upper figure shows the chopped DC current passing through the microwave stripline. Middle figure plots the corresponding ODMR spectra of the NV center in the hybrid nanothermometer. The sudden shift of the ODMR frequency is caused by the magnetic field from the chopped DC current. The lower figure is the temperature variation of the hybrid sensor under heating by the chopped current. (b) Heating and cooling dynamics measured by the hybrid nanothermometer. The $\Delta T = 0$ point is defined by the average of the data at the steady state of the heating/cooling process.

The hybrid nanothermometer has potential applications in monitoring thermal dynamics in microscopic systems such as biological thermal processes and heat dissipation in micro-/nano-electronic devices. For a proof-of-principle experiment, we utilized a microwave antenna around the hybrid nanothermometer as a heating source. We coupled a chopped DC current into the microwave stripline (which has a width of $20{\rm{\,\,\mu m}}$ and is located $\sim\!25{\rm{\,\,\mu m}}$ away from the sensor) using an RF/DC combiner. The heat generation/dissipation dynamics were monitored by real-time tracking of the local temperature at the location of hybrid nanothermometer. The chopped DC current is illustrated in the upper panel of Fig. 4a, and the corresponding ODMR signal is shown in the middle panel. When the DC current was chopped, an instantaneous change in the magnetic field induced by the DC current resulted in a sudden jump of the spin resonance frequencies in the ODMR, while heating and cooling processes were observed as evidenced by the subsequent spin resonance frequency shift after the DC current chopping. The temperature variation is plotted in the lower panel of Fig 4a, in which the ${\rm{\delta }}T\,\, = \,\,0\,\,$is arbitrarily defined. Figure 4b shows the temperature evolution (averaged over five chopping cycles). A temperature increase/decrease of 10 mK with a characteristic timescale $\sim\!1{\rm{\,\,s}}$ was clearly observed. In principle, the timescales are determined by several parameters such as the thermal contact between the diamond nanopillar and the antenna, distance between the hybrid sensor and the heating source, and the dissipation rate from the system to the environment. No delay was observed in the heating process, which means that the heat propagation/conduction time from the microwave stripline to the sensor was too short to be resolved in the measurements. The fast heat propagation (with timescale $\sim\!1\,\,{\rm{\mu s}}$) was a result of the high thermal conductivity of the bulk diamond ($ >\!\! 2200\,\,{\rm{W\,\,}}{{\rm{m}}^{ - 1}}{\rm{\,\,}}{{\rm{K}}^{ - 1}}$) and the short distance ($ \approx\! 30\,\,{\rm{\mu m}}$) between the heater and the sensor. The observation about the thermal dynamics was verified by a control experiment where alternating current with forward and reverse directions was applied with constant heating power. No frequency shift was observed following the jump caused by the electric magnetic fields (see Supplementary Fig. 7 for more details). This demonstration experiment proves the potential of the hybrid nanothermometer as a diagnostic tool for studying the thermal dissipation in microelectronics with high spatial and temperature resolution.

## DISCUSSION AND CONCLUSION

In conclusion, we developed an ultra-sensitive hybrid nanothermometer composed of a single NV center in a diamond nanopillar and a magnetic nanoparticle. When the environmental temperature changed near the critical temperature of the MNP, the magnetic field generated by the MNP abruptly changed. The magnetic field change was readily measured by the ODMR of the NV center. The sensitivity of the hybrid nanothermometer is as high as $76{\rm{\,\,\mu K\,\,H}}{{\rm{z}}^{ - 1/2}}$. The high temperature sensitivity indicates fast data acquisition, yet with a high temperature measurement precision. We applied the sensor to monitor the environment fluctuations as well as the *in situ* heat dissipation dynamics. Stable environmental temperature and large dynamic range are critical for further exploration of our hybrid nanothermometer to measure small temperature variation in systems of interest. In fact, the dynamic range of the hybrid sensor can be further enhanced by the frequency-locking scheme for the NV magnetometry [33].

To further improve the sensitivity of the hybrid sensor, we can increase the temperature susceptibility of the NV center resonance frequency. A 4-fold enhancement of the susceptibility is predicted theoretically by using a 200 nm diameter MNP and locating an NV center 25 nm from the MNP [28]. In addition, longer coherence time $T_2^*$ will improve the sensitivity as $\sim\!1/\sqrt {T_2^*} $ [34]. The dephasing time $T_2^*$ can be increased to 90 μs through use of an NV center in isotopically purified diamond [22,35], which means a 7-fold improvement of sensitivity. The performance of the nanosensors depends on their specific configuration and therefore each nanosensor needs to be characterized individually before measurement is carried out in realistic systems. To make the performance of the hybrid sensors (see Fig. 1 and Supplementary Fig. 6) more reproducible, one can employ MNPs with uniform size and composition [36] and control the MNP proximity to the NV center with AFM manipulation. Another method to stabilize the performance is to coat a magnetic alloy thin film (e.g., by thermal evaporation deposition) on diamond pillars with controllable thickness and composition. A further improvement of the hybrid nano-thermometer design is to replace the diamond nanopillar with a diamond cantilever [37,38] so that a scanning nano-thermometer can be realized with high spatial resolution. Such a design would allow extension of the present prototypical study of the heat dynamics induced by currents in microwave striplines (Fig. 4) to applications in realistic microelectric devices. An array of the hybrid nano-thermometers may also be constructed to measure the spatial distribution of temperature.

The ultra-sensitive hybrid nanothermometer is especially useful in measuring millikelvin temperature variation with high temporal resolution. Compared with existing ultrasensitive nano-thermometers (such as the SQUID-based nano-thermometer [17,18]), the hybrid diamond nano-thermometer features the applicability in ambient conditions. The new sensor may facilitate the study of a broad range of thermal processes, such as nanoscale chemical reactions, nano-plasmonics, heat dissipation in nano-/micro-electronics and thermal processes in single cells. As specific examples, the hybrid diamond nano-thermometer may be applied to investigation of thermal conduction and dissipation of nanowires and 2D materials [39], to measure fast (at millisecond timescale) temperature change during neuronal firing in neuron cells, and to detect small thermal gradient ($\sim\!{\rm{\mu K}}$ across a cell) inside live cells in different live stages [4].

## METHODS

### Experimental setup

A confocal-AFM correlation microscope was constructed to enable nano-manipulation of single copper-nickel alloy MNPs and *in situ* temperature measurements (see Supplementary Fig. 1). The AFM scanning head (BioScope Resolve, Bruker) was mounted on the confocal microscope to measure the topography and perform nanomanipulation of the MNP. The ODMR measurements were carried out using a home-built laser scanning confocal microscope. A $532{\rm{\,\,nm}}$ laser was adopted (MGL-III-532-200 mW, CNI) to excite the NV centers. An oil immersion objective lens (Nikon 100 × 1.45NA) was used to collect the NV’s fluorescence signal, which was then detected by an avalanche photodiode (APD, SPCM-AQRH-15-FC, Excelitas) and counted by data acquisition (DAQ, PCIe-6363, National Instruments). A microwave (MW) source (N5171B EXG Signal Generator, Keysight) and an amplifier (ZHL-16W-43-S+, Mini-Circuits) were used to generate microwave frequencies for spin measurements. A 20$\,\,{\rm{\mu }}$m copper wire was used to deliver MW. The sample temperature was controlled by a ceramic heater. The heating area of the heater is about $22\,\, \times {\rm{\,\,}}22\,\,$mm^2^ and the pillar was placed in the center with diamond membrane size of $1\,\, \times {\rm{\,\,}}1$ mm^2^. Considering the excellent thermal conductivity of diamond material, we assumed that the temperature was uniform across the diamond membrane (the distance between reference and hybrid sensor is also below 10${\rm{\,\,\mu m}}$). For details see Supplementary data.

### NV centers in diamond pillars

In the experiments, high fluorescence intensity of single NV centers in diamond can enhance the sensitivity of the hybrid nanothermometer. A tapered nanopillar shape diamond waveguide was fabricated to achieve enhancement of the fluorescence collection efficiency of single NV centers in diamond. The fabrication process was developed and introduced by Momenzadeh *et al.* [30], where the diamond waveguide was fabricated by electron beam writing and reactive ion etching processes. The optical and spin coherent properties of the NV center are shown in Supplementary Fig. 2. For details see Supplementary data.

### Temperature measurement and sensitivity estimation

The FID signal between the $|{m_s} = \,\,0\rangle $ and $|{m_s} = \,\, - 1\rangle $ states of the NV center was measured for estimation of the temperature sensitivity (method is illustrated in Fig. 2b). The FID signal, that is the photon count recorded at the end of the FID sequence [34], is

(2) \begin{equation*}S\!\left( t \right) \approx 1 - \frac{C}{2} + \frac{C}{2}\cos\! \left( {2\pi ft} \right)\exp\! \left[ { - {{\left( {\frac{t}{{T_2^*}}} \right)}^\nu }} \right], \end{equation*}

where $C$ is the contrast, $f\,\, = {f_{\rm{r}}}\,\, - {f_{\rm{p}}}$ is the detuning of the transition frequency ${f_{\rm{r}}}$ from the frequency of the microwave pulses ${f_{\rm{p}}}$, $T_2^*$ is the decoherence time and $\nu $ is the exponent of the decay. Least-square fitting of the FID signal (Fig. 2b) yielded the parameters $C\,\, = \,\,0.27$, $\delta f\,\, = \,\,2.7\,\,{\rm{MHz}},{\rm{\,\,}}T_2^* = \,\,1.8\,\,{\rm{\mu s}}$ and $\nu \,\, = \,\,3.3$.

The optimal evolution time ${t_{\rm{e}}}$ was determined by maximizing $| {\textit{dS}( {{t_{\rm{e}}}} )/{\textit{df}_{\rm{r}}}} |$. The FID signal variation depends on the temperature variation through

(3) \begin{equation*}\delta S\,\, = \frac{{dS\left( {{t_{\rm{e}}}} \right)}}{{d{f_{\rm{r}}}}}\,\, \times \frac{{d{f_{\rm{r}}}}}{{dT}} \times \delta T, \end{equation*}

where ${\textit{df}_{\rm{r}}}/dT$ is the temperature susceptibility (inset of Fig. 1d). Shot-noise of the FID signal per unit measurement time is $\sigma \approx 1/\sqrt {{L_{{\rm{eff}}}}} $, where ${L_{{\rm{eff}}}}$ is the effective photon count rate (including only the photons recorded during the readout time, which occupies about 10% of the whole FID duty cycle in Fig. 2b). The shot-noise limited sensitivity of the hybrid nanothermometer [34] is given by

(4) \begin{equation*} {\eta _T} = \,\,\sigma {\left| {\frac{{dS\left( {{t_{\rm{e}}}} \right)}}{{d{f_{\rm{r}}}}}} \right|^{ - 1}}{\left| {\frac{{d{f_{\rm{r}}}}}{{dT}}} \right|^{ - 1}}. \end{equation*}

Using the optimal temperature-susceptibility of the NV resonance frequency ${\textit{df}_{\rm{r}}}/dT\,\, = \,\,47\,\,{\rm{MHz\,\,}}{{\rm{K}}^{ - 1}}$ (see Fig. 1d) and the optimal evolution time ${t_{\rm{e}}} = \,\,1\,\,{\rm{\mu s}}$, and using the effective count rate ${L_{{\rm{eff}}}} = \,\,9.6\,\, \times {10^4}\,\,{{\rm{s}}^{ - 1}}$ (corresponding to a saturated count rate $\sim\!1\,\, \times {10^6}\,\,{{\rm{s}}^{ - 1}}$), we obtained the shot-noise limited temperature sensitivity to be ${\eta _T} \approx 76\,\,{\rm{\mu K\,\,H}}{{\rm{z}}^{ - 1/2}}$.

## ASSOCIATED CONTENT

Detailed description of the sample fabrication and experiment is available free of charge in the supporting information.

## Supplementary Material

nwaa194_Supplemental_File
