# Supplementary material for: Ultra-sensitive hybrid diamond nanothermometer
Source: Natl Sci Rev. 2020 Aug 28;8(5):nwaa194. doi: 10.1093/nsr/nwaa194 (PMC8288462; doi:10.1093/nsr/nwaa194)
Supplement: nwaa194_Supplemental_File [file nwaa194_supplemental_file.pdf]

Supplementary Information

of

Ultra-sensitive hybrid diamond nanothermometer

C.-F. Liu, et. al

Supplementary Note

Experimental setup

We constructed a microscope with correlated functions of confocal microscopy and atomic force microscopy (AFM), to enable nanomanipulation of single copper-nickel alloy magnetic nanoparticles (MNP) and in-situ temperature sensing measurements (see Supplementary Fig. 1). The optically detected magnetic resonance (ODMR) measurements were carried out using a home-built laser scanning confocal microscope. A 532 nm laser was used (MGL-III-532-200 mW, CNI), and a suppression of its power fluctuation down to 0.1% was realized by applying a PID feedback control. An oil immersion objective lens (Nikon 100x 1.45NA) was used to collect the fluorescence of single nitrogen-vacancy (NV) centers in the diamond pillars, which was then detected by an avalanche photodiode (APD, SPCM-AQRH-15-FC, Excelitas) and counted by a data acquisition card (DAQ, PCIe-6363, National Instrument). A pair of APDs were used to measure the second order correlation function of the fluorescence (to check whether the NV centers are good single quantum emitters).

A microwave (MW) source (N5171B EXG Signal Generator, Keysight), a microwave switch (ZASWA_2-50DR+Mini-Circuits), and an amplifier (ZHL-16W-43-S+, Mini-Circuits) were used to generate microwave pulses for spin measurements. A copper wire of width 20 μm was used to deliver the MW. The copper wire, when conducting a DC current, was also used as a heat source in the heat dissipation measurement.

The AFM scanning head (BioScope Resolve, Bruker) was mounted on the confocal microscope to measure the topography and to perform nanomanipulation of MNPs. We first acquired surface topographic images using the AFM tapping mode to localize the positions of the diamond pillars and copper-nickel alloy nanoparticles. The nanomanipulation was achieved by pushing the nanoparticles in the contact mode of the AFM. We re-imaged the surface to check the new positions of the MNPs and acquired the ODMR spectra to evaluate the coupling between the MNPs and the NV centers. Several manipulation processes were applied to optimize the coupling and finally we acquired the surface topography to record the positions of the MNPs and the pillars.

A vibration shielding was built to isolate the correlated microscope from the external environment using sound-absorbing foams. A temperature PID control module was used to keep the temperature fluctuation to < 0.05 °C. The local environment temperature was controlled by a ceramic heater, mounted on top of the glass sample holder.

NV centers in diamond pillars

In the experiments, high fluorescence intensity of single NV centers in diamond is required to enhance the sensitivity of the hybrid nanothermometer. Tapered nanopillar shape diamond waveguides were fabricated to improve the fluorescence collection efficiency of single NV centers in diamond. The fabrication process was developed and introduced by S. Momenzadeh et al [1]: the diamond waveguides were fabricated by electron beam writing and reaction ion etching processes. The optical and spin coherent properties of the NV center we studied for the nanothermometer is shown in Supplementary Fig. 2. The second order correlation function of the fluorescence from the pillar $g^{(2)}(\tau)$ is <0.5 at zero delay time (see Supplementary Fig. 2a), which indicates that the studied nanopillar contains only one single NV center. Supplementary Figure 2b plots the saturation curve of the single NV center. Under the excitation of $400\ \mu\mathrm{W}$ power, the saturated fluorescence counts of the single NV center was $1\ \mathrm{M\,s^{-1}}$. The spin resonance width of the NV center obtained from the ODMR spectra (Supplementary Fig. 2c) is consistent with the dephasing time under free induced decay (FID) measurement, $T_2^* = 1.5\ \mu\mathrm{s}$ (Supplementary Fig. 2d).

Temperature calibration

In the experiment, we calibrated the environment temperature by reference NV centers that were not affected by copper-nickel alloy MNPs (see Supplementary Fig. 3). We first performed the AFM scan to determine that no MNPs were next to the reference NV centers. We hence measured the ODMR spectra. There are four peaks in the ODMR spectra, which correspond to the the $m_s = \pm 1$ electron spin states and the nuclear spin hyperfine coupling. The resonance frequencies were extracted by multi peaks Lorentzian fitting of the ODMR spectra and the zero-field splitting (D) was deduced by taking the mean value of the resonance frequencies. Supplementary Fig. 3b shows the ODMR spectra of the reference NV centers for different heating currents of the ceramic heater. We determined the environment temperatures corresponding to the heating current of the ceramic heater using the coefficient as $\mathrm{d}D/\mathrm{d}T = -74\ \mathrm{kHz\,K^{-1}}$ (see Supplementary Fig. 3c).

Reversibility of the nanothermometer in cooling/heating cycles

We performed heating and cooling processes of the hybrid nanothermometer several times, and recorded the ODMR spectra of the sensor. The spin resonance frequencies of the sensor at different temperature and the corresponding temperature susceptibility for the additional measurements are plotted in Supplementary Fig. 4. Compared with the results in the main text, the sensor shows same spin resonance frequencies during the heating and cooling processes, indicating good reversibility of the hybrid nanothermometer.

Sensitivity estimation of a few other hybrid nanothermometers

We have also fabricated two more hybrid nanothermometers composed of single copper-nickel alloy MNPs and single NV centers in diamond nanopillars.

For a nanopillar we detected a single NV center with high fluorescence intensity and long dephasing time, we pushed a single MNP next to the pillar. The AFM images of the hybrid nanothermometers after the AFM nano-manipulation (see insets of Supplementary Figs. 6a and 6d) show that the MNPs were positioned next to the diamond nanopillars. We further measured the spin resonance frequencies of the sensors as functions of the temperature (see Supplementary Figs. 6a and 6d). The temperature susceptibility of each sensor is plotted in Supplementary Figs.

6b and 6e. At the ferromagnetic-paramagnetic transition temperature, the temperature susceptibilities were optimal, being $10\ \mathrm{MHz\ K^{-1}}$ or $7\ \mathrm{MHz\ K^{-1}}$. The NV center spin dephasing times in free induction decay (FID) of the two sensors were measured to be $3.5\ \mu\mathrm{s}$ and $1.6\ \mu\mathrm{s}$ (see Supplementary Figs. 6c and 6f). Using the measured dephasing times $3.5\ \mu\mathrm{s}$ and $1.6\ \mu\mathrm{s}$, we estimate that these two hybrid nanothermometers have optimal temperature sensitivities of $230\ \mu\mathrm{K\ Hz^{-1/2}}$ and $550\ \mu\mathrm{K\ Hz^{-1/2}}$, respectively.

Temperature sensing study of heat generation and dissipation

We applied the hybrid nanothermometer to sense the thermal dissipation during heating by a DC current conducted by the copper wire. To confirm that the resonance frequency shifts after the jumping were caused by the temperature dynamics (see Fig. 4 in the manuscript). , we ran the current in both positive and negative directions (the electrical heating effect was maintained the same) and monitored the thermal dissipation by the hybrid nanothermometer (see Supplementary Fig. 7a). In the beginning, we applied the current I_o to the microwave antenna and the current direction was controlled by a microcontroller. When the current through the copper wire switched its direction, a jump of the ODMR spectra was caused by the change of the magnetic field, as seen in Supplementary Fig. 7b. The temperature variation for positive current (Supplementary Fig. 7c) and that for negative current (Supplementary Fig. 7d) present no difference within measurement precision, which confirms that the resonance frequency shift in the manuscript was mainly caused by the temperature variation induced by the pulsed heating current through the copper wire.

Reference

[1] S. A. Momenzadeh, R. J. Stöhr, F. F. deOliveira, A. Brunner, A. Denisenko, S. Yang, F. Reinhard, J. Wrachtrup, “Nanoengineered Diamond Waveguide as a Robust Bright Platform for Nanomagnetometry Using Shallow Nitrogen Vacancy Centers,” Nano Lett. **15**, 165–169 (2015).

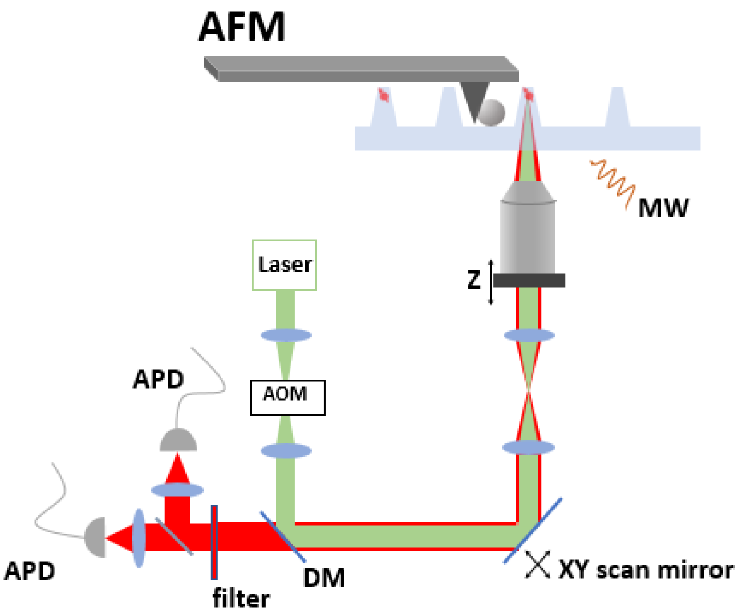

Supplementary Figure 1. Experimental setup. APD: Avalanche photon diode. AOM: acoustic optical modulate. DM: dichroic mirror. AFM: atomic force microscopy. MW: Microwave.

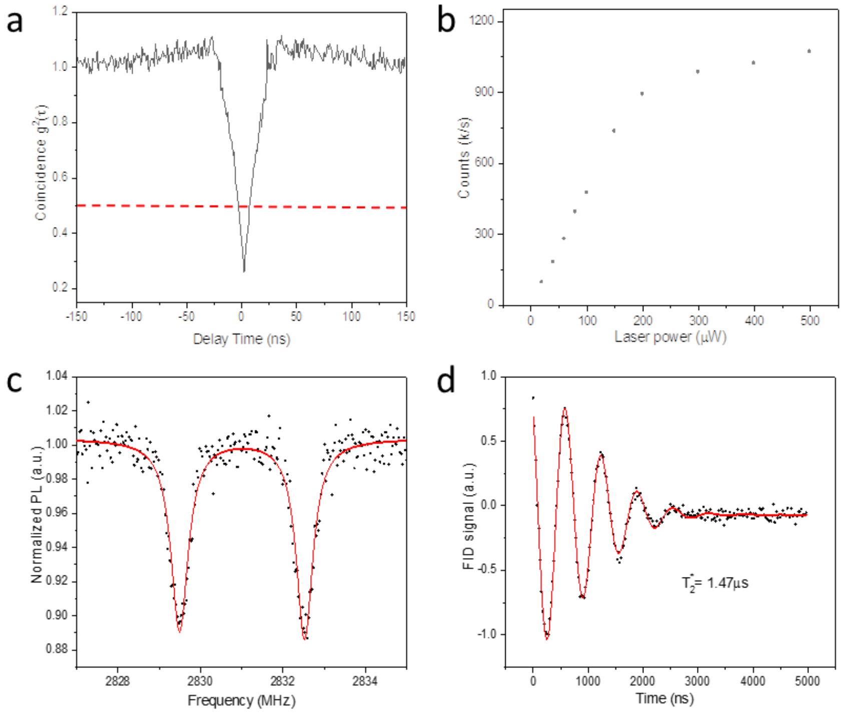

Supplementary Figure 2. Optical and spin properties of the NV center for the nano-thermometer in main text. (a) Normalized second order correlation function $g^{(2)}(\tau)$ of the fluorescence of the center. (b) Optical saturation behavior of the single NV center. (c) Pulse ODMR spectrum of the NV center under an external magnetic field, showing the ^{15}N hyperfine coupling and narrow linewidths. (d) FID of the single NV center with dephasing time $T_2^* = 1.47\ \mu s$.

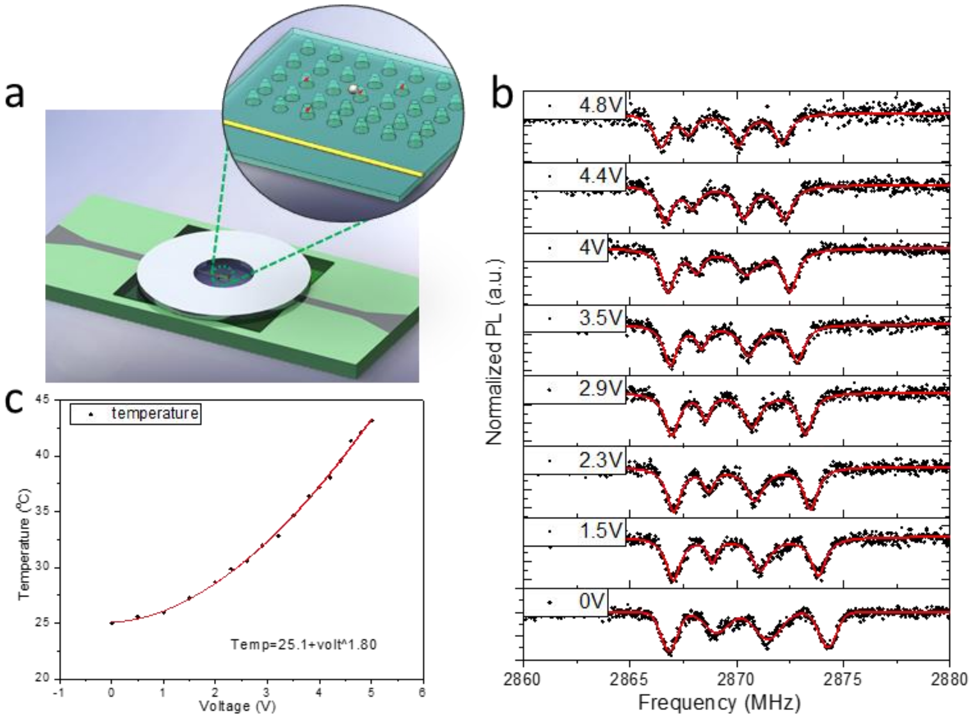

Supplementary Figure 3. Temperature calibration of the systems. (a) Setup for temperature sensing. The ceramic heater is on top of the diamond sample (white plate). (b) Typical ODMR spectra of a single NV center in the diamond nanopillar under zero external magnetic field (without MNPs in the proximity) for different ceramic heater voltages. (c) Calculated environment temperature as a function of the voltage applied to the ceramic heater.

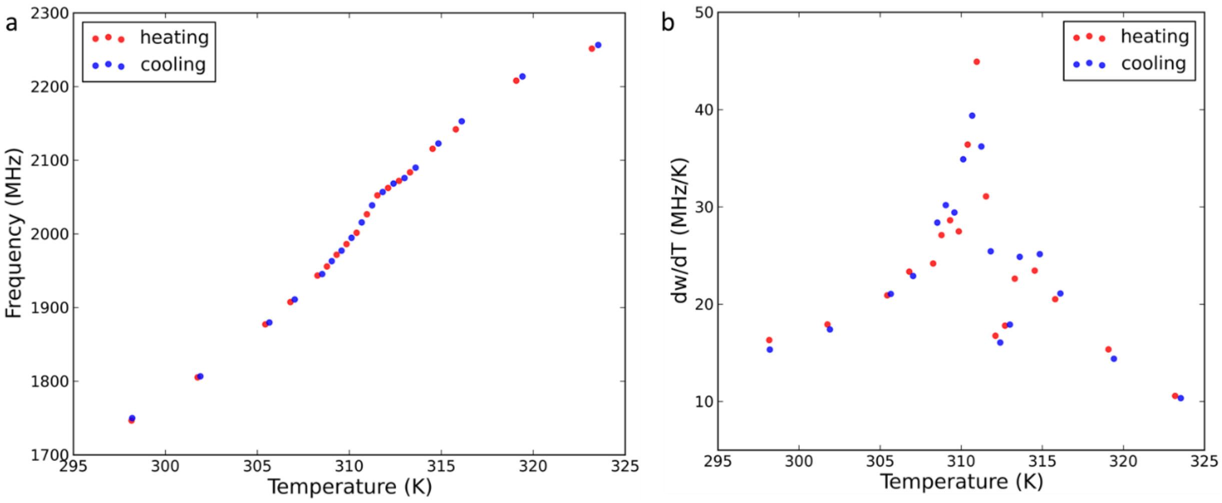

Supplementary Figure 4. Reversibility of the hybrid nanothermometer. (a) Spin resonance frequencies of the NV center in the hybrid nanothermometer during heating and cooling processes (additional to those present in the main text). (b) Temperature susceptibilities of the hybrid nanothermometer versus the temperature in the additional cooling and heating processes.

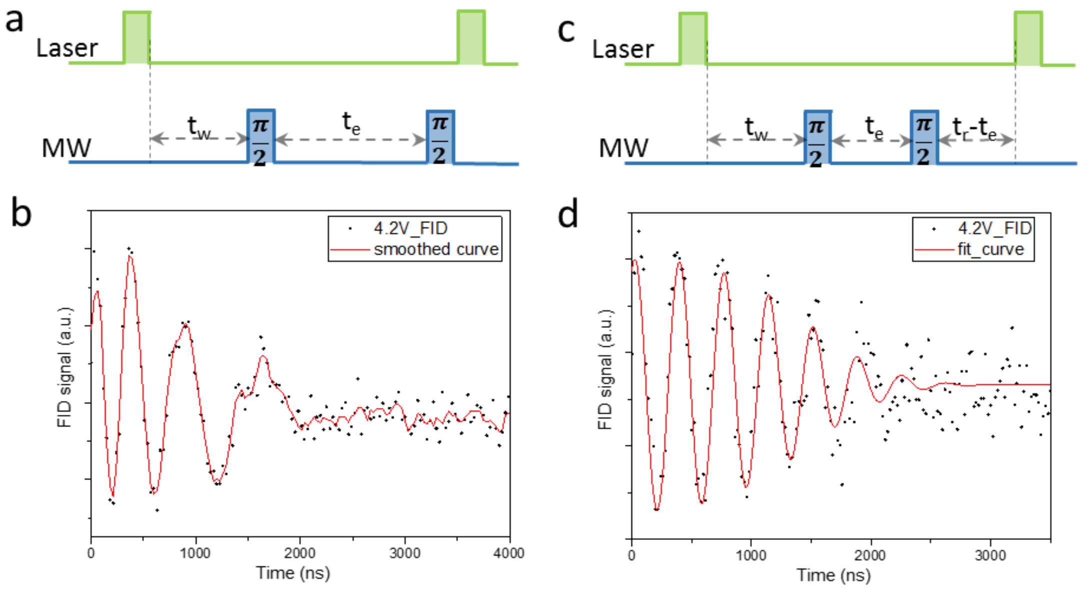

Supplementary Figure 5. FID signal with and without laser heating duty ratio control. (a) Pulse sequence of the measurement and (b) FID signal without laser heating ratio control. (c) Pulse sequence and (d) FID signal with laser heating control.

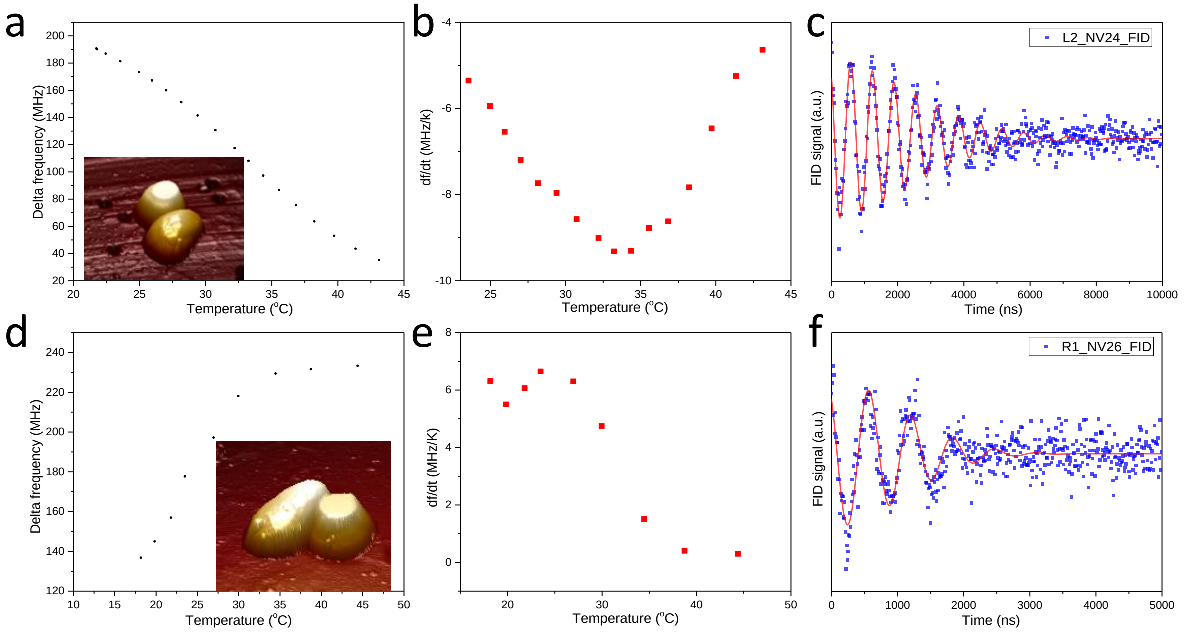

Supplementary Figure 6. Two additional hybrid sensors. (a) Spin resonance frequencies of the 2nd hybrid nanothermometer versus environment temperature. Inset is the atomic force microscopy (AFM) image of the 2nd hybrid sensor. (b) Temperature susceptibility of the 2nd hybrid nanothermometer versus environment temperature. (c) Free induction decay (FID) of the 2nd hybrid sensor, $T_2^* = 3.5\ \mu s$. (d) Spin resonance frequencies of the 3rd hybrid nanothermometer versus environment temperature. (e) Temperature susceptibility of the 3rd hybrid nanothermometer versus environment temperature. (f) FID of the 3rd hybrid sensor, $T_2^* = 1.6\ \mu s$.

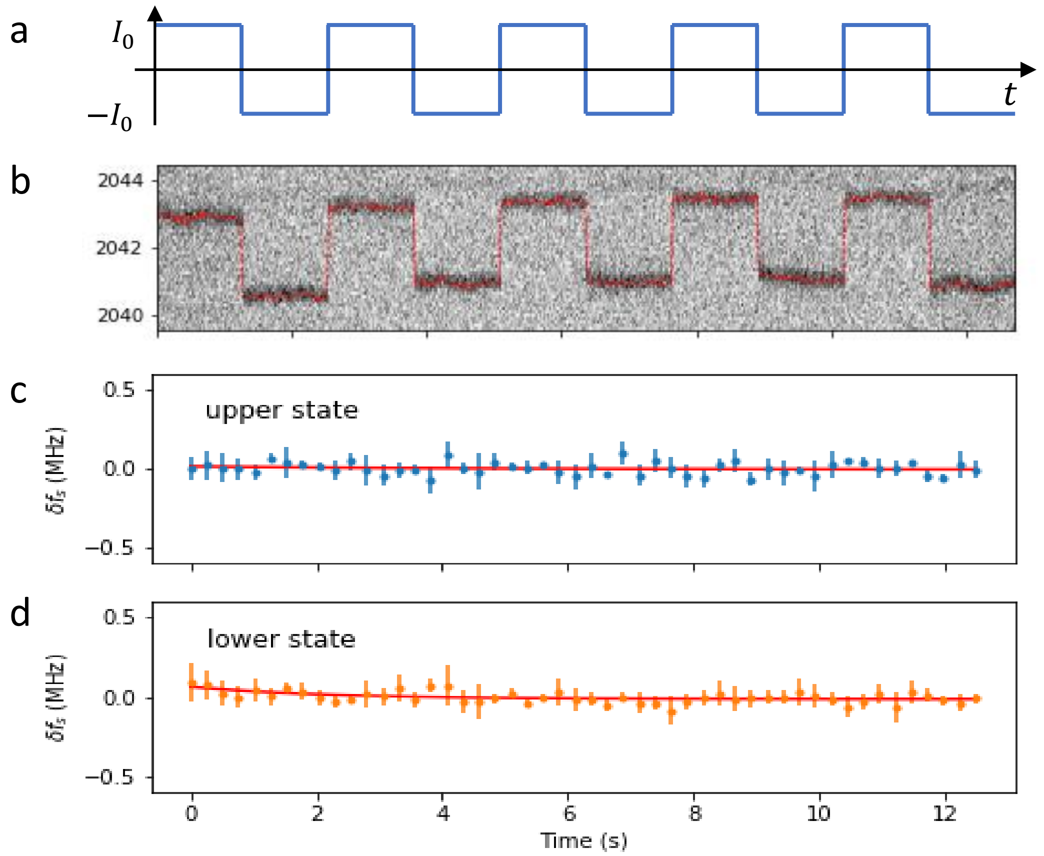

Supplementary Figure 7. Heating by two opposite currents through the copper wire. (a) Schematic of the current sequence. (b) ODMR spectra of the hybrid nanothermometer when the current was running through the wire. (c) and (d) Sensed resonance frequency variationwhen the current was positive and negative, respectively.
